# Functional Assessment and Coordination Characteristics of Production, Living, Ecological Function—A Case Study of Henan Province, China

**DOI:** 10.3390/ijerph18158051

**Published:** 2021-07-29

**Authors:** Jianchun Fu, Shaoliang Zhang

**Affiliations:** 1 School of Environment Science and Spatial Informatics, China University of Mining and Technology, Xuzhou 221116, China; fjc@hpu.edu.cn; 2 School of Surveying and Land Information Engineering, Henan Polytechnic University, Jiaozuo 454000, China; 3 Research Centre of Arable Land Protection and Urban-Rural High-Quality, Development of Yellow River Basin, Jiaozuo 454000, China

**Keywords:** territory space, PLE function, coordination feature, spatial autocorrelation, Henan Province, China

## Abstract

Multifunctional state assessment was the basis of time sequence design of territory spatial development and overall utilisation. This study aimed to identify the ecological-production-living (PLE) territory spatial function to provide a basis for territory spatial planning. It took Henan Province as the research area. This study developed a methodology to assess differentiation characteristics for PLE function, a method that integrates functional merging and geographic information technology. We used the coordination degree model and spatial autocorrelation analysis to reveal the coordination of spatial functions of the province. The results were as follows: (1) During the study period, the land production function of main grain-producing areas decreased slowly, and production and living function values of the Central Plains urban agglomeration with Zhengzhou as the centre were in an upward trend. The characteristics of urban-rural dualization were prominent, and ecological function value decreased year by year. (2) The laws of territorial spatial functions had different manifestations in different stages (1990–2005 and 2005–2018). By different characteristic laws, the change in production function in the later period was bigger than that in the previous period. The living function maintained a good continuity expansion characteristic before and after. The spatial regularity distribution characteristics of ecological function were weak, and the overall environment became worse than before. (3) The territory space of middle and low coordination function types was the most important type, and the aggregation was relatively weak. Xuchang County and Weihui City showed better states of functional coordination aggregation. Lushi County, Xinxian County, and Shangcheng County, which were in the western and southern mountainous and hilly areas, showed low-low aggregation characteristics. Thus, the government will strengthen targeted control over territorial space. This study provides a reference for the overall deployment of the development and utilisation of territory space in Henan Province.

## 1. Introduction

The concept of the production, living, and ecological (PLE) spaces originated from the report of the 18th National Congress of the Communist Party of China. It emphasises establishing the territory spatial development patterns in the functional perspective. The PLE spaces correspond to the three zones and three lines of the territory spatial development planning. The proposal of this concept can promote the construction of reasonable ecological, production, and life security patterns [1,2]. The difference in the territory spatial functions of the PLE spaces has greatly reflected the economic structural levels. With the acceleration of industrialisation and urbanisation in China, the conflict of spatial functions is becoming more and more motivated [3,4]. This situation leads to the escalation of the game of land use types. It affects the ecological environment, investment environment, and human settlement environment [5,6,7]. Therefore, assessing the territorial spatial functions can reveal the characteristic law of functional evolution and guide the management and planning of territorial space in the new era.

Functional diversity and functional complexity are the essential attributes of land resources. These properties have inspired scholars to explore the scientific issues related to the function of territorial space [8,9,10,11].

In the academic circle, there is still no unified definition of PLE functional. Generally speaking, it contains the following three aspects. First of all, the production function is related to the industrial structure, pursuing intensive land use and efficient output. Secondly, the service objects of life functions are mainly person, who pursue the convenience and livability of life [12,13]. Third, the better the ecological conditions, the higher the degree of ecosystem functions of the land space. On the contrary, the worse the ecosystem environment quality conditions, the worse it will hinder the performance of ecosystem functions [14,15]. The functional identification method is divided into a quantisation measuring algorithm and a merges classification method [16,17]. The quantisation calculation algorithm quantitatively identifies the PLE functions of the territorial space. Jin et al. [18] selected the index factors representing different thematic elements of the territory space from the perspective of the PLE functions. He constructed an index system based on “element-function” to evaluate the secondary functions of territory space in the Wuhan urban agglomeration of China. However, huge disparities existed among the different regions or units of different scales of the evaluation systems and methods. Thus, the method is hard to use in practice and has uncertainty.

The essence of the merging classification method is to merge and classify land-use types. This method can compensate for the deficiency of land use classification by considering ecological functions to a certain extent and is easy to connect with other land-use classification systems [19]. Liu [2] merged the territory production, living, and ecological space service functions in China based on the dialectical relationship between land use types and functions, land use classification standards, and the principle of ‘bottom-up, functional grading’. However, the diversity of territorial spatial functions leads to uncertainty in assessment [20,21]. In a word, the evaluation of territorial spatial function should comprehensively consider the actual regional characteristics of the research object, the accessibility of data, and the need for ease of operation [22,23].

Henan Province is a traditional agricultural province in China. After the reform and opening-up policy, industrialisation and urbanisation are developing rapidly, and the spatial structure and function of its territory have constantly changed. This condition has gradually revealed some problems, such as unreasonable territory development sequence, unscientific spatial layout, and improvement of urban-rural integration [24,25]. Therefore, the key to compiling territorial spatial planning is to accurately identify the ‘function’ of the territorial space carrying and service characteristics [26]. The local people’s government is implementing the three national strategies of China (Henan): the Pilot Free Trade Zone, the Central Plains Economic Zone, and the Zhengzhou Airport Economy Zone. Henan Province will support the new opportunities for economic development. The rational development and utilisation of land and resources in Henan Province are conducive to realise national strategic goals.

This study evaluated the characteristics of the PLE function of territorial space in Henan Province, supporting remote sensing monitoring data of land use in 1990, 2005, and 2018. It further reveals the coordination state between functions to provide decision supports for territory spatial planning in the new era.

## 2. Geography and Methods

### 2.1. Study Area

**Figure 1:**
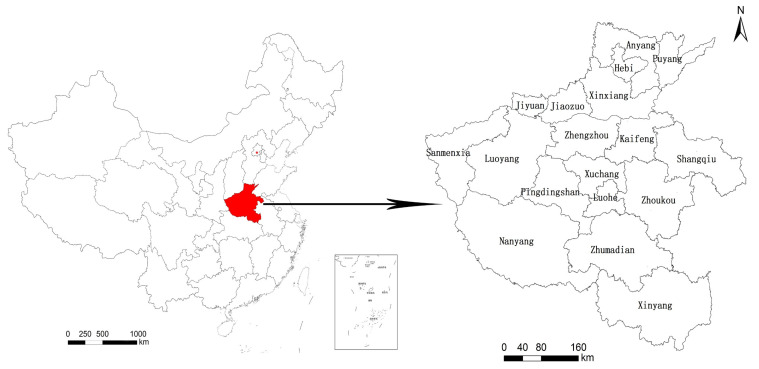
Administrative area map of Henan Province.

Henan Province (31°23′–36°22′ N, 110°21′–116°39′ E) is located in the central and eastern parts of China and the middle and lower reaches of the Yellow River (Figure 1). It has jurisdiction over 18 provincial cities, such as Zhengzhou, Kaifeng, Luoyang, and Pingdingshan, 20 county-level cities, and 65,700 km^2^. Its climate belongs to the transition zone of the warm temperate with the continental monsoon climate zone. The spring is dry and the summer is rainy. The terrain is high in the west and low in the east. The Taihang Mountains, the Funiu Mountains, the Tongbai Mountains, and the Dabie Mountains show a semi-annular distribution along the provincial boundary. The Huang-Huai-Hai alluvial plain lies in the middle and east, and the Nanyang Basin lies in the southwest of Henan. Henan Province is the most populous and agricultural province in China. It occupies an important position in the supply of commodity grain in China. At present, Henan Province is in the stage of accelerated industrialisation development. Its urbanisation rate is below 70% of the industrialisation rate. There is a large gap between urbanization regions. The speed of natural ecological protection and restoration is relatively backward, and the territory ecological security is facing a great threat.

### 2.2. Data Sources

**Figure 2:**
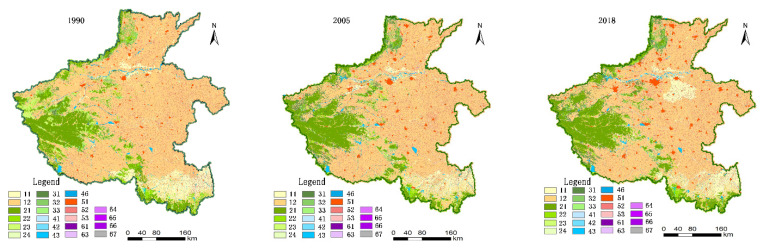
Land use maps in 1990, 2005, and 2018 of Henan Province.

The land uses remote sensing monitoring data from the Chinese Academy of Sciences Resources and Environment Science Data Centre (http://www.resdc.cn, accessed on 12 October 2020) which are the production data generated by artificial visual interpretation [27]. The primary data sources are the Landsat TM/ETM 30 m resolution remote sensing images from June to September. Land-use types include six primary types of cultivated land, forest land, grassland, water area, residential land, unused land, and 25 secondary types. The total classification accuracy was over 94.31%. Many researchers applied these remote sensing images for land-use change and dynamic remote sensing monitoring due to their high precision and practicability. We formed the data of land use status in Henan Province in 1990, 2005, and 2018 after coordinate system conversion, cropping, and other processes of the acquired remote sensing monitoring data (Figure 2 and Table 1).

### 2.3. Methods

#### 2.3.1. Evaluation of the PLE Functions of Territorial Space

Functional diversity and complexity are the basic attributes of territorial resources. Based on the difference in land structure, according to the perspectives of the industrial, social, and ecological management attributes of regional land use, this study divided the land use type into three types: production, living, and ecological land in sequence. The concepts of strong-PLE land, semi-PLE land, and weak-PLE land were introduced according to the big and weak differences of land use versatility. Based on differences in the strength of land use versatility, an evaluation model of PLE function for different land-use types was constructed [28]. The researchers proposed an assignment rule according to different types of land use (strong-PLE functions assignment 5, semi-PLE functions assignment 3, weak-PLE functions assignment 1, and no-PLE functions assignment 0) (Table 1). This study uses the assigning method to evaluate the PLE function characteristics of territorial space.

This study draws on the grid sampling method in land-use change analysis to break the constraints of land-use types. It takes the average patch area of 2–5 times of geographical grid as an example (800 m × 800 m) as the regional unit of PLE functional assessment. We superimposed these grids with the current land use and calculated the corresponding land type and area in each geographic grid. It calculated the function value of each geographic grid based on the area of each region for the functional assessment. The spatial distribution map is generated by the inverse distance weight interpolation method [29,30].

(1) $$F_{i}=\overset{n}{\underset{i=1}{\sum }}S_{i}\times W_{i}$$

*F_i_* is the PLE function value of each geographic grid. *i* is the land-use type. *n* is the total number of land-use types in each geographic grid. *S_i_* is the area of the *i*th land-use type in the geographic grid. *W_i_* is the PLE function assigned by unit area of the *i*th land-use type in the geographic grid.

#### 2.3.2. Characteristics of the Functional Differentiation of Territorial Space

This study investigates the differentiation characteristics of the PLE functions of territorial space from two aspects: the spatial distribution within the year and the interannual dynamic change.

(1) It analyses the spatial distribution characteristics of the PLE functions of the territorial space during the year by a qualitative description. (2) The natural breaks classification (NBC) [31] method obtains feature points following the statistical law of natural values, which maximise the difference between different grades [32]. The PLE functions in 1990–2005 and 2005–2018 are calculated through grid computing on the ArcGIS platform. The difference between the two times is analysed through grid evaluation. The three types of change are divided into ‘shrink’, ‘maintain’, and ‘expand’, based on the NBC method. Geographical information system (GIS) space statistical techniques are to analyse the characteristics of the changes. This process reflects the dynamic change in the territorial PLE functions of the interannual characteristics.

#### 2.3.3. Coordination Features of Territory Spatial Functions

This study builds a model of the degree of coordination of territorial space based on the research of relevant scholars [33]. This model evaluates the coordination degree of territorial spatial functions from the perspectives of production, living, and ecology. It also identifies the coordinated development level of the functions of production, living, and ecology quantitatively.

(2) $$D=\sqrt{{\left\{\frac{f_{1}\times f_{2}\times f_{3}}{\left(f_{1}+f_{2}\right)\times \left(f_{1}+f_{3}\right)\times \left(f_{2}+f_{3}\right)}\right\}}^{1/3}\times (\alpha f_{1}+\beta f_{2}+\gamma f_{3)}}$$

*D* is the coordination degree of territorial functions. The greater the value of *D*, the better the coordination degree. The degree of coordination reflects the level of harmony between production, living, and ecological function systems in the development process. *f*1, *f*2, and *f*3 correspond to the indices of land production, living, and ecological functions. *A*, *β*, and *γ* are undetermined coefficients. $\alpha f_{1}+\beta f_{2}+\gamma f_{3}$ constituted the comprehensive function index of the territory. The virtuous cycle and development of the territorial space are closely related to the guaranteed realisation degree of any of these functions.

#### 2.3.4. Spatial Autocorrelation Characteristics

Spatial autocorrelation reflects some type of phenomenon in an area and adjacent area unit on the relevance of the same phenomenon. It can reveal the potential dependence degree between land spatial function coordination [34]. Spatial autocorrelation includes global spatial autocorrelation and local spatial autocorrelation. (1) Global spatial autocorrelation can measure the average level of the entire study area’s functions of territorial space coordination in terms of Moran’s I index and the range of −1 < *I* ≤ 1 or less. If the value is less than 0, the overall characteristics are negatively correlated. If the value is equal to 0, the overall characteristics are irrelevant. If the value is greater than 0, the spatial correlation is relatively large, and the clustering characteristics are strikingly apparent. The specific data formula is

(3) $$I=\frac{n\sum_{i=1}^{N}\sum_{i=1}^{N}w_{ij}(x_{i}-\bar{x})(x_{j}-\bar{x})}{S_{0}\sum_{i=1}^{N}{\left(x_{i}-\bar{x}\right)}_{}^{2}}$$

where *n* represents the number of county units. *x_i_* is the observed value (in this study, the observed value of the spatial unit is the sampling unit in the county). $\bar{x}$ is the mean value of *x_i_*. $S_{0}=\sum_{i=1}^{N}\sum_{i=1}^{N}w_{ij}$. *w_ij_* is the spatial connection matrix between the research objects *i* and *j*. When *i* and *j* are adjacent spatial relations, *w_ij_* = 1. Conversely, *w_ij_* = 0. Moran’s I index needs to be tested for significance, and Z-value is used for testing. When *Z* < −1.96 or *Z* > +1.96, *p* < 0.05, that is, the confidence coefficient is greater than 95% and can be considered significant.

(2) Researchers measure local spatial autocorrelation by local indicators of spatial association. The local indicators of the spatial association are adopted to reflect the degree of correlation (positive correlation) or difference (negative correlation) between the attributes of a certain county unit and its surrounding county units and visualise them spatially.

(4) $$I_{i}=\frac{\left(x_{i}-\bar{x}\right)}{S^{2}}\sum_{j}w_{ij}(x_{j}-\bar{x})$$

*x_i_*, *x_j_*, and *w_ij_* have the same meanings as above, and *S*^2^ represents the variance of the observation unit of *x_j_*. An *I_i_* value greater than 0 indicates the spatial aggregation of similar values (high or low values) around the unit in this region. An *I_i_* value of less than 0 indicates the spatial aggregation of no similar values. The study takes the mean value of the coordination degree of territorial spatial function within the county as an observation variable and then standardizes these variables. Based on the Queen rule, this study analyses the spatial autocorrelation of coordination degree of territorial spatial functions in Henan Province.

## 3. Results and Analysis

### 3.1. Distribution Characteristics of PLE Functions in Territorial Space within the Year

Henan Province is the most important agricultural and industrial province in China. On the one hand, its grain output ranks at the forefront of all provinces, and it is the most significant grain production base and commodity export base in China. It played a critical role in ensuring the country’s food security. On the other hand, the economy is growing very fast, and its total GDP ranks first in the middle and western regions. The above-mentioned good agricultural and industrial conditions affect the temporal and spatial characteristics of the land production function.

**Figure 3:**
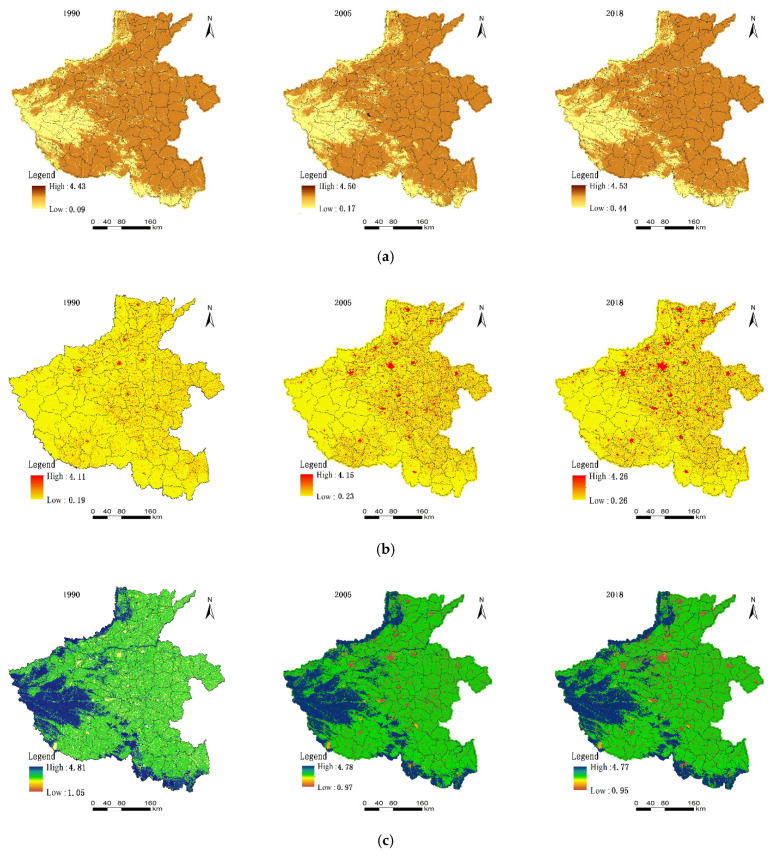
Function evaluation value of territorial space of Henan Province. (**a**) Function evaluation value of production; (**b**) function evaluation value of living; and (**c**) function evaluation value of ecological.

The territorial spatial functions maintain a relatively stable state in Henan Province. (Figure 3). The mean value of production function gradually increased to 2.30, 2.32 and 2.33, respectively in 1990, 2005 and 2018. This finding reflects that land production function, land-intensive, and high output efficiency are enhanced. This condition presents that the spatial distribution characteristics in the western mountains have a low function value, whereas the eastern plain has a high value.

The Chinese government has put forward relevant regional strategic planning such as the Development Plan of Central Plains Urban Agglomeration, the Promotion of the Rise of Central China during the 13th Five-Year Plan, and Central Plains Urban Agglomeration. These plannings have become a new economic growth engine, and the centre of national production gradually shifting to the central and northern parts of Henan province. Rapid economic development promotes the continuous improvement of residents’ living standards. The average values of a living function at the three research time points were 0.48, 0.53, and 0.58, respectively. Residents’ life is convenient, and the space is liveable.

The living function of territorial space developed steadily in a benign direction. This condition showed that the function value in the western is relatively low, and the high value in the eastern is scattered. The mean values of territory ecological functions are 3.17, 3.14, and 3.09. The ecological service function of territorial space has continued to decline in the past 28 years. This condition is due to the area of forest and grassland caused by economic production and construction decreases year by year. Henan Province has gradually constructed Tongbai Dabie mountain ecological zones, Funiu mountain ecological zones, South Taihang ecological zones, and plain ecological conservation zones. The local government has also built the ecological conservation belt of the Yellow River beach area across the east and the west, and the environment protection belt of the middle route of the South-to-North Water Transfer Project across the north and the south. The regional ecological security strategic pattern of Four Zones and Two Belts was constructed. These events have a significant positive impact on the territory ecological space of Henan Province.

### 3.2. Interannual Variation Characteristics of the PLE Functions of Territorial Space

Authors calculate the difference between the raster evaluation results at different time points and analyse the change characteristics of shrinking, maintaining, and expanding of the functions in the previous period (1990–2005) and the later period (2005–2018). This process reflected the difference and phased characteristics of the interannual variation characteristics of the PLE functions of territorial space.

#### 3.2.1. Production Function

Economic development has entered a stage of rapid development of varying magnitudes since the reform and opening of Henan Province in the last century. It gradually shifted from a planned economy to a market economy, and to an industrialised economy since the late 1980s. Local governments have eliminated several inefficient mines and industries around major cities. This condition reflects directly on the land production function. In the previous period (1990–2005), the production function of territorial space in Henan Province was shrinking, and this phenomenon mainly concentrated in the surrounding areas of Zhengzhou City. The proportion of the territorial area with shrinking characteristics reached 13.43%, and the region of functional expansion accounted for 2.36%.

**Figure 4:**
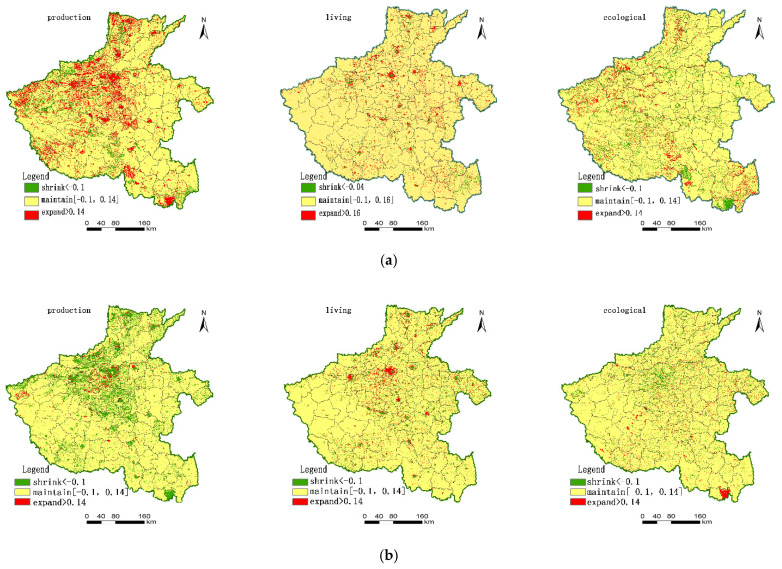
Changes of PLE functions of territory space in Henan Province. (**a**) Changes of functions from 1990 to 2005; (**b**) changes of functions from 2005 to 2018.

After China acceded to the WTO in 2002, Henan’s economy began to enter a period of multi-polarity-driven global development and developed in an all-round way. In the latter period (2005–2018), the change in territorial spatial production function was significantly bigger than in the previous period. The production function changes in the eastern plain were mostly in the stable state, accounting for 80.05%, and approximately 14.55% of the territory was in the expansion state. The expansion area mainly concentrated in the central and northern regions, part of the western and southern regions, and 5.39% of the territory was shrinking production function. The characteristic difference of the production function is especially evident, and the main reason is that the characteristics of the economic development of Henan Province are different in different periods (Figure 4).

#### 3.2.2. Living Function

The phased economic development characteristics also reflect in the changes in living function. In the previous period (1990–2005), many migrant workers in Henan Province entered the city, and the urbanisation process showed the characteristics of urban expansion around central cities such as Zhengzhou and Luoyang. Expansion of living function was the main change feature of the pattern, with an area proportion of 3.49%, and mainly concentrated in the central, eastern, and northern regions. Most of the functionally stable areas are southern region, accounting for 92.18%. The area of functional reduction was 4.34%. The living functional areas were concentrated mostly in the central and northern regions, which are the political and economic centres of Henan Province (Figure 4). In the latter period, the functional expansion area concentrated in the central and northern regions, with an area proportion of 4.53%. This area maintained a good continuity and expanded sharply compared with the previous period. The relative area proportion of functional reduction area was 4.51%, and the area proportion of functional stability area was 90.96%. In the latter period, the living functional areas continued to shrink and agglomerate. The area increased, and the urbanisation process of Henan Province continued to advance.

#### 3.2.3. Ecological Function

The characteristics of large population and small land have made the ecological environment the main factor affecting sustainable development. For a long time, sustained economic development has made the local ecological environment gradually deteriorate.

In the previous period (1990–2005), the expansion and contraction of ecological functions coexisted, and the regularity of spatial distribution was weak. The area statistics showed that the area proportion of ecological function expansion was 5.54%. The area of functional reduction area was larger than that of the ecological expansion area, with the proportion reaching 6.70%. During this period, the area of high ecological function area decreased, and the area proportion of functional stability area reached 87.76%. During this period, the local government paid more attention to economic development issues, while the ecological and environmental problems did not attract enough attention.

In the latter period (2005–2018), the area proportion of functional expansion area was 3.69%. The area proportion of functional reduction area was 5.14%, and the area proportion of functional stability area increased to 91.17% (Figure 4). Compared with the previous period, the ecological function index gradually decreased. In the national economic development, the emphasis on economic construction and the neglect of the ecological environment made the local ecological environment develop in a negative direction. In the future, the ecological function should focus on the optimal allocation of territorial space.

### 3.3. Analysis of the Coordination Characteristics of PLE Functions in Territorial Space

Territorial space has the characteristics of dynamic evolution, which is reflective of the corresponding changes in the function. The transformation of economic and social development has shifted the development model of territorial space from production-oriented to integrated coordination of PLE. This synergy relationship is the mirror of the degree of coupling and coordination.

**Figure 5:**
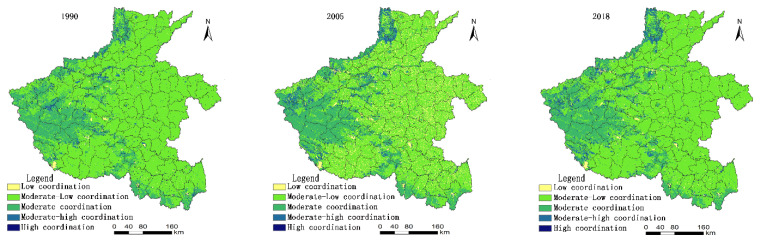
Evaluation results of coordination function of territory space in Henan Province from 1990 to 2018.

**Figure 6:**
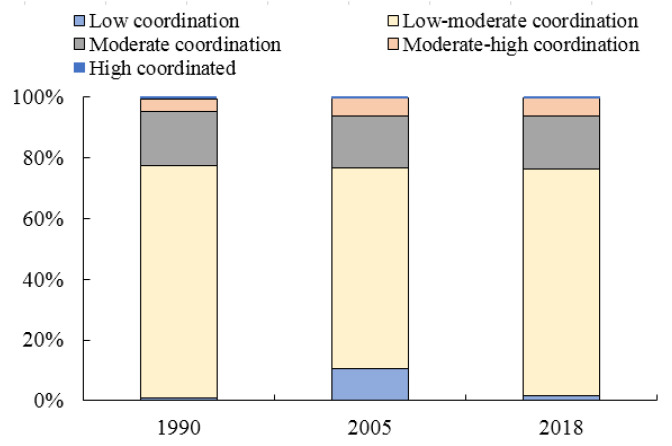
Histogram of evaluation results of land spatial function coordination degree in Henan Province from 1990 to 2018.

Based on the comprehensive evaluation results of the PLE functions of territorial space, this study evaluates the characteristics of land and space coordination in Henan Province. The natural split classification method was adopted to divide the grades (high, medium-high, medium-low, and low levels) to maximise the differences between grades (Figure 5 and Figure 6).

For a long time, the development model of some areas in Henan Province at the expense of the environment and the extensive use of resources has resulted in a large area of land in the middle and low coordination types. The area of middle and low coordination areas has increased with the new wave of rapid economic development.

In 1990, Henan Province is still in a period of extensive economy. Medium and low coordination accounted for the majority, with an area proportion of 76.33%, and this type of region was widely distributed. The second is the moderately coordinated type, with an area proportion of 17.93%. This type is predominant in the western, northern, and southern regions of Henan Province, and the geomorphic types were mainly mountainous and hilly.

In 2005, the range of change of middle-low degree coordination region was extremely large, and the proportion decreased by approximately ten percentage points.

However, the local government has begun to enter a period of rapid economic development, inefficient industrial land, heavy scale heavy quantity development mode prevailing. The area of low coordination degree increased by approximately ten percentage points. It is mainly in the central and eastern plain areas. The area of medium coordination degree increased by approximately two percentage points compared with 1990, with a small change of 17.08%. In 2005, the coordination degree of territorial function showed that the area of the intermediate coordination section decreased, whereas the region of the two-end increased. The characteristics of coordinated polarisation of territorial functions are distinct. In 2018, the local government had taken many measures in the intensive use of land, especially construction land, which has improved the coordination level of low-coordination land use to a certain extent. The proportion of territory areas with moderate and low coordination reached 75.01% with a fast growth rate. The proportion of territory areas with low coordination decreased to 1.67%, whereas the proportion of land area with moderate coordination remained stable, with an area proportion of 17.43%. Medium-high and high coordination areas are small, accounting for 5.73% and 0.50%, respectively (Figure 6).

### 3.4. Spatial Autocorrelation Analysis of Functional Coordination Degree

Moran’s I index reflects the average state of the coordination of territory spatial function in the whole study area. The larger the value of Moran’s I index, the better the overall spatial aggregation characteristics. The results show that the Moran’s I indices of spatial function coordination degree of each county in Henan Province in 1990, 2005, and 2018 are all greater than 0, which are 0.42, 0.30, and 0.30, respectively. The coordination degree of territorial spatial function at each research time point has significant spatial autocorrelation characteristics. There are potential interdependencies among the functional coordination degrees. The spatial correlation is strong. The aggregation characteristics are evident. However, the Moran’s I index in 2005 and 2018 is significantly lower than that in 1990, reflecting the regularity of the weakening of aggregation. The main reason is that it is a large traditional agricultural province, with stable agricultural development and high intensity. In the early stage of economic development, the Huang-Huai-Hai agricultural production zones located in the central and eastern regions showed more concentration. With the high-intensity development of the economy, the production, and living functional areas have gradual and scattered expansion characteristics, which weakened the clustering characteristics.

**Figure 7:**
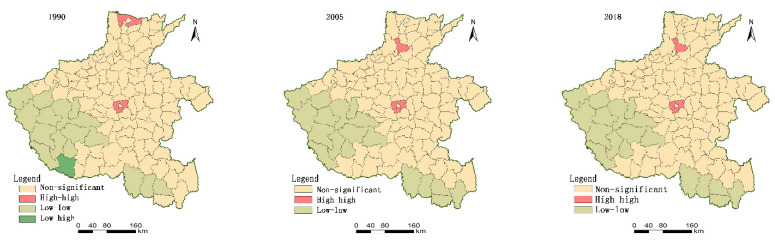
Local spatial autocorrelation graph of county dynamics in the territorial space of Henan from 1990 to 2018.

We measured the degree of functional coordination spatially by local spatial autocorrelation analysis. High-high aggregation characteristics indicate that the functional coordination degree of a single county and surrounding counties is high. The functional coordination degree of the territorial space is in a relatively stable state (Figure 7).

Therefore, this county should be regarded as the key for maintaining the PLE functions of the territorial space. In 1990, Anyang County and Xuchang County were the two counties with high-high functional coordination degrees. Weihui and Xuchang County are the two counties in 2005 and 2018. The characteristics of a low-low aggregation reflect that the coordination degree of territory spatial function in this county is at a low level, and the coordination degree of surrounding counties is at a low level. Affected by the function of assimilation, the county often requires considerable territory spatial control efforts. In 1990, the function of 16 counties, including Lushi, Ruyang County, Song County, Luanchuan County, and Lushan County was in a low-low coordination state of aggregation. Most of the above counties are located in the western and southern mountainous areas. The ecological condition of these counties is relatively good, and conditions of living and production are poor. This situation leads to poor coordination of territorial space functions. In 2005 and 2018, the function coordination degree of 18 counties is in a low-low aggregation state, and the spatial consistency is higher than that in 1990. Xin County and Shangcheng County are new low-low agglomeration counties, reflecting the weakening of functional coordination degree of neighbouring counties. Therefore, strengthening the territory spatial planning and comprehensive control of their land is necessary. For low-high aggregation counties, the territory spatial function coordination degree is in a low position. The coordination degree of surrounding counties is high. Therefore, this county can be regarded as the focused object of territorial spatial management. In 1990, only Dengzhou City’s function coordination degree was in the clustering state. However, this clustering type of counties disappeared by 2005 and 2018.

## 4. Discussion

The pattern of territorial space can reflect the level of the economic structure. In turn, economic development can promote the continuous change of the pattern. Although this change has played a fundamental role in supporting economic development, it has also brought about a series of problems such as unreasonable use of land, excessive concentration of population in some areas, drastic changes, and continuous deterioration in the ecological environment.

During the 40 years of reform and opening-up, China’s economy has made great achievements. The rapid development of industrialisation and urbanisation has brought intensive changes in territorial space. On the one hand, this change has promoted the rapid development of China’s economy. On the other hand, it has brought a series of unreasonable utilisation problems of territorial space. It is necessary to find ways to deal with the chaotic order of territory spatial development during the rapid economic development, the inefficiency of resource utilisation, and the gradual deterioration of the ecological environment. It is necessary to explore the changing characteristics of the spatial temporal pattern and the changing process of the functional also. After going through this series of measures, it can play a fundamental, strategic, and comprehensive role in the strategic management of territorial space.

Henan Province is the most important agricultural and industrial province, and its grain output ranks at the forefront of China. It is the most significant grain production base, and also the base of the output in China. It has played an important role in the protection of national grain security. The economic growth of Henan Province is extremely rapid. The territory spatial structure of the whole province has changed accordingly. It is necessary to reveal the characteristics of the spatial pattern of the territorial space function in the historical period of Henan Province. Therefore, it is significant for the formulation of spatial planning and regional strategy of Henan Province.

On the one hand, this study used the ‘land use remote sensing monitoring data’ interpreted by the Chinese Academy of Sciences, and these data are authoritative in China. Using these data to research territorial space function identification can effectively solve the problems mentioned above. On the other hand, the territorial space has the multifunction attribute, and the land use type corresponds to a certain function attribute. The essence of the research on territorial space is to study its corresponding function. Considering the revised evaluation of the territorial spatial function is a necessary process of territorial spatial function identification based on the existing research of territorial spatial identification. Therefore, the follow-up study should evaluate the territory spatial functions of Henan or other provinces through the function revision evaluations of the territory spatial PLE function. We should explore the pattern characteristics of the PLE function from the differentiation and coordination.

## 5. Conclusions

This study assessed the characteristics of the spatial PLE functions of Henan Province based on multi-period land use remote sensing data. Its pattern characteristics were analysed from the perspectives of differentiation and coordination. The main conclusions are as follows:

(1) During the study period, the value of territorial production function increased. The value of production function in the main grain-producing areas located in the eastern Huang-Huai-Hai Plain decreased slowly. The change in land production function is mainly reduced. Overall, the living function is lower in the west and higher in the east. The growth rate of urban living space is significantly higher than that of rural living space. The rural economic development speed is slow, but the growth rate of urban living space is fast, and the characteristics of urban-rural dualization are distinct. The ecological function values of Funiu Mountain in the west, South Taihang Mountain in the north, and Tongbai Dabie Mountain in the north are relatively high. However, the ecological environment and service functions of the region are gradually declining over time. Affected by the economic development strategies of Henan Province at different stages, the pattern of land use has changed, and this is reflected in the changes in the spatial function. In the latter period (2005–2018), the change in production function is significantly better than the previous period (1990–2005). The living function in two periods maintains the characteristics of continuous expansion. The spatial regularity distribution of ecological function was weak, and the overall environmental conditions become worse than before;
(2) The system of territorial space is a complex system composed of multiple factors with PLE functions as its core function. The interaction and influence of the functions of PLE functions have made economic and social transformations. The transformation of economic and social development has shifted the development model of territorial space from production-oriented to integrated coordination of PLE. This synergy relationship is the mirror of the degree of coupling and coordination. For a long time, the development model of some areas in Henan Province at the expense of the environment and the extensive use of resources has resulted in a large area of land in the middle and low coordination types. With the new wave of rapid economic development, the area of middle and low coordination areas has increased;
(3) The status of regional differences in functional coordination is vital to formulate regional strategies for territorial spatial planning. For a long time, the agglomeration of territory spatial functions was weak, but still shows outstanding agglomeration characteristics. High-high and low-low are the main types of local autocorrelation. The two counties at different periods are all in the high-high clustering state. The low-low clustering state of Lushi County, Ruyang County, Xinxian County, and Shangcheng County is mainly in the western and southern mountainous areas of Henan Province. Therefore, these regions need to strengthen the territory spatial control.

## Figures and Tables

**Table 1:** Evaluation assignment table of territorial spatial functions.

Land Use Classification System	Production Function	Living Function	Ecological Function	Land Use Classification System	Production Function	Living Function	Ecological Function
11 (Irrigated field)	3	0	3	43 (Reservoir and pond)	1	0	1
12 (Dry land)	3	0	3	46 (Beach land)	0	0	5
21 (Forestland)	1	0	5	51 (Urban land)	5	5	0
22 (Shrub land)	0	0	5	52 (Rural settlements)	5	5	0
23 (Open wood land)	0	0	5	53 (Other construction lands)	5	0	0
24 (Other woodlands)	0	0	3	61 (Sandy land)	0	0	1
31 (High-coverage meadow)	1	0	5	63 (Gobi)	0	0	1
32 (Medium-coverage meadow)	1	0	5	64 (Saline land)	0	0	1
33 (Low-coverage meadow)	1	0	3	65 (Bare land)	0	0	1
41 (river and canal) 42 (Lakes)	0 0	0 0	5 5	66 (rock land)	0	0	1
				67 sup(Other land)	0	0	1

## Data Availability

Not applicable.

## References

[1] Tian F., Li M., Han X., Liu H., Mo B. (2020). A Production–Living–Ecological Space Model for Land-Use Optimisation: A case study of the core Tumen River region in China. Ecol. Model..

[2] Liu J., Liu Y., Li Y. (2017). Classification evaluation and spatial-temporal analysis of ‘production-living-ecological’ spaces in China. Geogr. Sci..

[3] Lin G., Jiang D., Fu J., Cao C., Zhang D. (2020). Spatial Conflict of Production–Living–Ecological Space and Sustainable-Development Scenario Simulation in Yangtze River Delta Agglomerations. Sustainability.

[4] Zou L., Liu Y., Yang J., Yang S., Wang Y., Cao Z., Hu X. (2020). Quantitative identification and spatial analysis of land use ecological-production-living functions in rural areas on China’s southeast coast. Habitat Int..

[5] Baja S., Pulubuhu D.A.T., Neswati R., Arif S. (2019). Nurmiaty Land Use Conflict with a Particular Reference to Spatial Planning Implementation in South Sulawesi. IOP Conf. Ser. Earth Environ. Sci..

[6] Lema M.A., Majule A.E. (2009). Impacts of climate change, variability and adaptation strategies on agriculture in semi-arid areas of Tanzania: The case of Manyoni District in Singida Region, Tanzania. Afr. Environ. Sci. Technol..

[7] Zou L., Liu Y., Wang J., Yang Y., Wang Y. (2019). Land use conflict identification and sustainable development scenario simulation on China’s southeast coast. Clean. Prod..

[8] Duh J.D., Daniel G.B. (2007). Knowledge-informed Pareto simulated annealing for multiobjective spatial allocation. Comput. Environ. Urban. Syst..

[9] Aslan T.A., Kirmikil M., Gündoğdu K.S., Arici I. (2018). Reallocation model for land consolidation based on landowners’ requests. Land Use Policy.

[10] Sifa O., Harun U., Huseyin H. (2021). Implementation of meta-heuristic optimization algorithms for interview problem in land consolidation: A case study in Konya/Turkey. Land Use Policy.

[11] Tomal M. (2021). Evaluation of coupling coordination degree and convergence behaviour of local development: A spatiotemporal analysis of all Polish municipalities over the period 2003–2019. Sustain. Cities Soc..

[12] Godschalk D.R. (2004). Land use planning challenges-coping with conflicts in visions of sustainable development and livable communities. J. Am. Plan. Assoc..

[13] Daur N., Adam Y.O., Pretzsch J. (2016). A historical political ecology of forest access and use in Sudan: Implications for sustainable rural livelihoods. Land Use Policy.

[14] Duinker P.N., Greig L.A. (2007). Scenario analysis in environmental impact assessment: Improving explorations of the future. Environ. Impact Assess. Rev..

[15] Stein E.D., Doughty C.L., Lowe J., Cooper M., Sloane E.B., Bram D.L. (2020). Establishing Targets for Regional Coastal Wetland Restoration Planning Using Historical Ecology and Future Scenario Analysis: The Past, Present, Future Approach. Estuar. Coasts.

[16] Yang Y., Bao W., Liu Y. (2020). Coupling coordination analysis of rural production-living-ecological space in the Beijing-Tianjin-Hebei region. Ecol. Indic..

[17] Sharafatmandrad M., Khosravi Mashizi A. (2021). Investigating tradeoffs between supply, use and demand of ecosystem services and their effective drivers for sustainable environmental management. J. Environ. Manag..

[18] Brown G., Glanz H. (2018). Identifying potential NIMBY and YIMBY effects in general land use planning and zoning. Applied. Geogr..

[19] Bengochea D., Henderson K., Loreau M. (2020). Agricultural land use and the sustainability of social-ecological systems. Ecol. Mod..

[20] Maria L.P., Cesare P.M., Laurence M., Marta P.S. (2011). An aggregation framework to link indicators associated with multifunctional land use to the stakeholder evaluation of policy options. Ecol. Indic..

[21] Lu L., Zhou S., Zhou B. (2013). Land use transformation and its eco-environmental response in process of the regional development: A case study of Jiangsu Province. Sci. Geogr. Sin..

[22] Yu Z., Xu E., Zhang H., Shang E. (2020). Spatio-Temporal Coordination and Conflict of Production-Living-Ecology Land Functions in the Beijing-Tianjin-Hebei Region, China. Land.

[23] DeFries R.S., Foley J.A., Asner G.P. (2004). Land-use choices: Balancing human needs and ecosystem function. Front. Ecol. Environ..

[24] Lin J., Liu X., Xu H., Liu Y., Liu S. (2010). Uneven growth and regional sustainable development-research on the development structure of Henan Province. Resourc. Environ. Yangtze Basin.

[25] Guo Y., Zhang C., Kang Y. (2015). Land assessment division research on economic development in Henan province. Geogr. Res..

[26] Brown G., Raymond C. (2014). Methods for identifying land use conflict potential using participatory mapping. Landsc. Urban Plan..

[27] Ning J., Liu J., Kuang W., Xu X., Zhang S., Yan C., Li R., Wu S., Hu Y., Du G. (2018). Spatiotemporal patterns and characteristics of land-use change in China during 2010–2015. J. Geogr. Sci..

[28] Bai R.S., Jiang Y.P., Jiang J.D. (2016). Analyzing spatial distribution patterns of ecological-production-living spaces of Jianghuai Urban Agglomeration. China Anc. City..

[29] Tunçay T., Bayramin I., Atalay F., Ünver I. (2016). Assessment of Inverse Distance Weighting (IDW) Interpolation on Spatial Variability of Selected Soil Properties in the Cukurova Plain. Tarım Bilim. Derg..

[30] Sui H., Song G., Zhang H. (2020). Identification of production-living-ecological space at Keshan County level in main grain-producing areas in northern Songnen Plain, China. Trans. Chin. Soc. Agric. Eng..

[31] Garcia A.S., Vilela V.M.d.F.N., Rizzo R., West P., Gerber J.S., Engstrom P.M., Ballester M.V.R. (2019). Assessing land use/cover dynamics and exploring drivers in the Amazon’s arc of deforestation through a hierarchical, multi-scale and multi-temporal classification approach. Remote Sens. Appl. Soc. Environ..

[32] Tian P., Li J., Shi X., Wang L., Liu R. (2018). Spatial and temporal changes of land use pattern and ecological risk assessment in Zhejiang Province. Resourc. Environ. Yangtze Basin.

[33] Shan W., Jin X., Ran N., Fan Y., Liu J., Zhou Y. (2019). ‘Production-Living-Ecological’ function evaluation and coupling coordination analysis of land use in Jiangsu Province. Resourc. Environ. Yangtze Basin.

[34] Moctezuma V. (2021). Spatial autocorrelation in a Mexican dung beetle ensemble: Implications for biodiversity assessment and monitoring. Ecol. Indic..

